# Geographic and environmental impacts on gut microbiome in Himalayan langurs (*Semnopithecus schistaceus*) and Xizang macaques (*Macaca mulatta vestita*)

**DOI:** 10.3389/fmicb.2024.1452101

**Published:** 2024-09-04

**Authors:** Xueyu Wang, Hong Li, Yumin Yang, Zhijiu Wu, Zhixiang Wang, Dayong Li, Wancai Xia, Shuzhen Zou, Yujia Liu, Fan Wang

**Affiliations:** ^1^Key Laboratory of Conservation Biology of Rhinopithecus roxellana (Department of Education of Sichuan Province), China West Normal University, Nanchong, China; ^2^Institute of Agricultural Resources and Environment, Sichuan Academy of Agricultural Sciences, Chengdu, China; ^3^Affiliated Hospital of North Sichuan Medical College, Nanchong, China

**Keywords:** gut microbiomes, geographic and environmental impacts, functional adaptations, Himalayan langurs (*Semnopithecus schistaceus*), Xizang macaques (*Macaca mulatta vestita*)

## Abstract

**Introduction:**

Gut microbiome plays a crucial role in the health of wild animals. Their structural and functional properties not only reflect the host’s dietary habits and habitat conditions but also provide essential support for ecological adaptation in various environments.

**Methods:**

This study investigated the gut microbiome of Himalayan langurs (*Semnopithecus schistaceus*) and Xizang macaques (*Macaca mulatta vestita*) across different geographic regions using 16S rRNA gene and metagenomic sequencing.

**Results:**

Results showed distinct clustering patterns in gut microbiota based on geographic location. Soil had an insignificant impact on host gut microbiome. Himalayan langurs from mid-altitude regions exhibited higher levels of antibiotic resistance genes associated with multidrug resistance, while Xizang macaques from high-altitude regions showed a broader range of resistance genes. Variations in carbohydrate-active enzymes and KEGG pathways indicated unique metabolic adaptations to different environments.

**Discussion:**

These findings provide valuable insights into the health and conservation of these primates and the broader implications of microbial ecology and functional adaptations in extreme conditions.

## Introduction

The gut microbiome plays a crucial role in host health, influencing processes such as digestion, metabolism, immune function, and resistance to pathogens (Wu et al., 2021; Colella et al., 2023). Various factors, including diet, genetics, and environmental conditions, shape the composition and functionality of the gut microbiome (Hasan and Yang, 2019; Franson et al., 2021; Moya-Alvarez and Sansonetti, 2022; Nova et al., 2022). Among these factors, the impact of geographic location and habitat conditions has been extensively documented. Studies have demonstrated significant differences in the gut microbiota of wild animals across various habit environment, including reptiles (Zhou et al., 2020), birds (Nagai et al., 2019), non-human primates (NHPs) (Ni et al., 2020; Sawaswong et al., 2021; Bornbusch et al., 2022a; Lan et al., 2022), herbivorous ungulates (Gao et al., 2019; Haworth et al., 2019; Gao et al., 2020; Meili et al., 2024), and carnivorous mammals (Wasimuddin Menke et al., 2017; Guo et al., 2019; Gillman et al., 2020; Ning et al., 2020). Geographic location and environmental factors, particularly high-altitude environments, are critical in determining the structure and function of gut microbial communities (Wang et al., 2022; Yang et al., 2022). High-altitude environments, such as those found in the Himalayas, are characterized by extreme conditions, including low oxygen levels, high ultraviolet radiation, and cold temperatures (Wang et al., 2024). These harsh conditions impose significant physiological stresses on resident animals, necessitating unique adaptations for survival. The gut microbiota is integral to these adaptations, facilitating various metabolic processes and contributing to overall health and resilience (Ma et al., 2019; Li et al., 2023). Previous studies on Qinghai-Xizang Plateau fish (*Glyptosternum maculatum*) (Pan et al., 2021), Xizang antelope (*Pantholops hodgsonii*) (Ma et al., 2019), and Xizang wild asses (*Equus kiang*) (Ma et al., 2019; Gao et al., 2020) in high-altitude regions have demonstrated significant ecological adaptations of their gut microbiota to these environments. These adaptations are often linked to the host’s dietary habits and environmental conditions, highlighting the crucial role of gut microbiota in ecological adaptation and metabolic function at high altitudes.

The Himalayan langurs (*Semnopithecus schistaceus*), also known as the Himalayan gray langur, belongs to the family Cercopithecidae and is found in regions of India, Nepal, Sri Lanka, and southern Xizang in China (Guo and Li, 2019). This species is the first-class protected wildlife in China, which is primarily arboreal but can also move on the ground and typically inhabits mid-altitude tropical rainforests, subtropical evergreen broadleaf forests, and mixed needle-broadleaf forests below 3,000 meters above sea level. They are known for their social behavior, often forming groups of several dozen individuals, and are most active during dawn and dusk (Perlman et al., 2016; Nautiyal et al., 2023). Their diet is omnivorous, consisting of leaves, wild fruits, and occasionally insects and small vertebrates. The Xizang macaques (*Macaca mulatta vestita*) is a subspecies of rhesus macaque of the second-class protected wildlife in China, which that inhabits regions above 3,000 meters in high altitude. This subspecies is primarily distributed in eastern and southern Xizang and northwestern Yunnan in China (Li et al., 2023). Xizang macaques live in high mountain forests and stone mountain gorges, feeding on buds, leaves, fruits, and seeds of alpine vegetation. Their adaptation to high-altitude environments makes them an excellent model for studying the gut microbiota’s response to different altitudes. The geographic and habitat differences between Himalayan langurs and Xizang macaques populations provide a unique opportunity to explore gut microbiota adaptations across different environment.

Environmental microbiome also plays a significant role in shaping host gut microbiota. Exposure to soil microbiota provides opportunities for horizontal bacterial transfer and the acquisition, potentially influencing the structure and function of the host gut microbiota (Blum et al., 2019; Anwar et al., 2021). Previous studies have demonstrated that soil environments can play a crucial role in facilitating the acquisition of nutrients and the horizontal transfer of microbiota among various NHPs. This phenomenon has been observed in species such as langurs (*Semnopithecus schistaceus*), ring-tailed lemurs (*Lemur catta*), Verreaux’s sifaka (*Propithecus verreauxi*), red-tailed sportive lemurs (*Lepilemur ruficaudatus*), and red-fronted brown lemurs (*Eulemur rufifrons*) (Monaco et al., 2019; Perofsky et al., 2019; Bornbusch et al., 2022b). For instance, the geophagy (Soil-eating behavior) observed in ring-tailed lemurs is believed to be linked to nutritional and microbial supplementation (Bornbusch et al., 2022b). Similarly, Nepalese gray langurs (*S. schistaceus*) have been found to ingest soil to obtain sodium (Monaco et al., 2019). These behaviors highlight the potential for soil environments to influence the gut microbiota of primates by providing essential nutrients or beneficial microbes. Moreover, even when primates are not directly consuming soil, they may still ingest soil microbiota indirectly while foraging on food that has fallen to the ground. Such findings contribute to expanding our understanding of how environmental factors mediate the composition and functional properties of primate gut microbiota.

Antibiotic resistance genes (ARGs) are a major concern for global public health (Larsson and Flach, 2022). These genes can be horizontally transferred between different bacterial species, facilitating the spread of resistance across various environments and hosts (Zainab et al., 2020; Tao et al., 2022). Studies have shown that different wild geographic populations of giant pandas exhibit distinct patterns in the distribution of ARGs (Hu et al., 2021). In wildlife, the presence and distribution of ARGs can offer valuable insights into how environmental factors and human activities contribute to the proliferation of antibiotic resistance in natural ecosystems (Laborda et al., 2022). Understanding the distribution and dynamics of ARGs in wildlife is crucial for assessing the potential risks posed by antibiotic resistance. In addition, carbohydrate-active enzymes (CAZymes) play a vital role in the degradation, modification, and synthesis of carbohydrates (Benini, 2020). These enzymes are essential for the digestion of complex polysaccharides found in the diet. It has been reported that the abundance of genes encoding microbe-produced enzymes involved in carbohydrate metabolism, particularly glycoside hydrolases, varies significantly among different geographic reintroduction sites for Przewalski’s horse (*Equus przewalskii*) in China (Tang et al., 2023). Understanding the diversity and function of CAZymes in the gut microbiota can shed light on how different species adapt to their dietary environments, especially in high-altitude regions where food sources may be limited and require specialized digestive capabilities.

In this study, we aim to explore the gut microbiota of Himalayan langurs and Xizang macaques living in different geographic and altitudinal regions. In designing our study, we specifically aimed to assess the influence of regional environmental factors on the gut microbiota of primates. Recognizing the importance of geographical and ecological diversity, we carefully selected sampling sites that are representative of broader ecological environments within the region. Although soil and fecal samples were not collected from identical locations, the chosen sampling areas reflect the wide-ranging environments these primates inhabit, allowing for a more comprehensive understanding of how different environmental factors influence gut microbiota across diverse landscapes. Based on fecal and soil samples, utilizing the full-length 16S rRNA gene sequencing and metagenomic sequencing, we seek to address two primary questions:(1) Does the soil environment significantly influence the gut microbiota of the two primates? (2) Do Himalayan langurs and Xizang macaques exhibit adaptive differences in their gut microbiota composition, ARGs, CAZymes, and KEGG pathways in response to varying ecological habitats? This study contributes to our understanding of how geographic and environmental factors influence the gut microbiota of Himalayan langurs and Xizang macaques, providing valuable insights into their health and conservation. Moreover, this study also could offer insights into the broader implications of microbial ecology and functional adaptations in high-altitude environments. These contributions advance the growing body of knowledge on the adaptability of gut microbiota under extreme conditions.

## Materials and methods

### Samples collection

**Figure 1:**
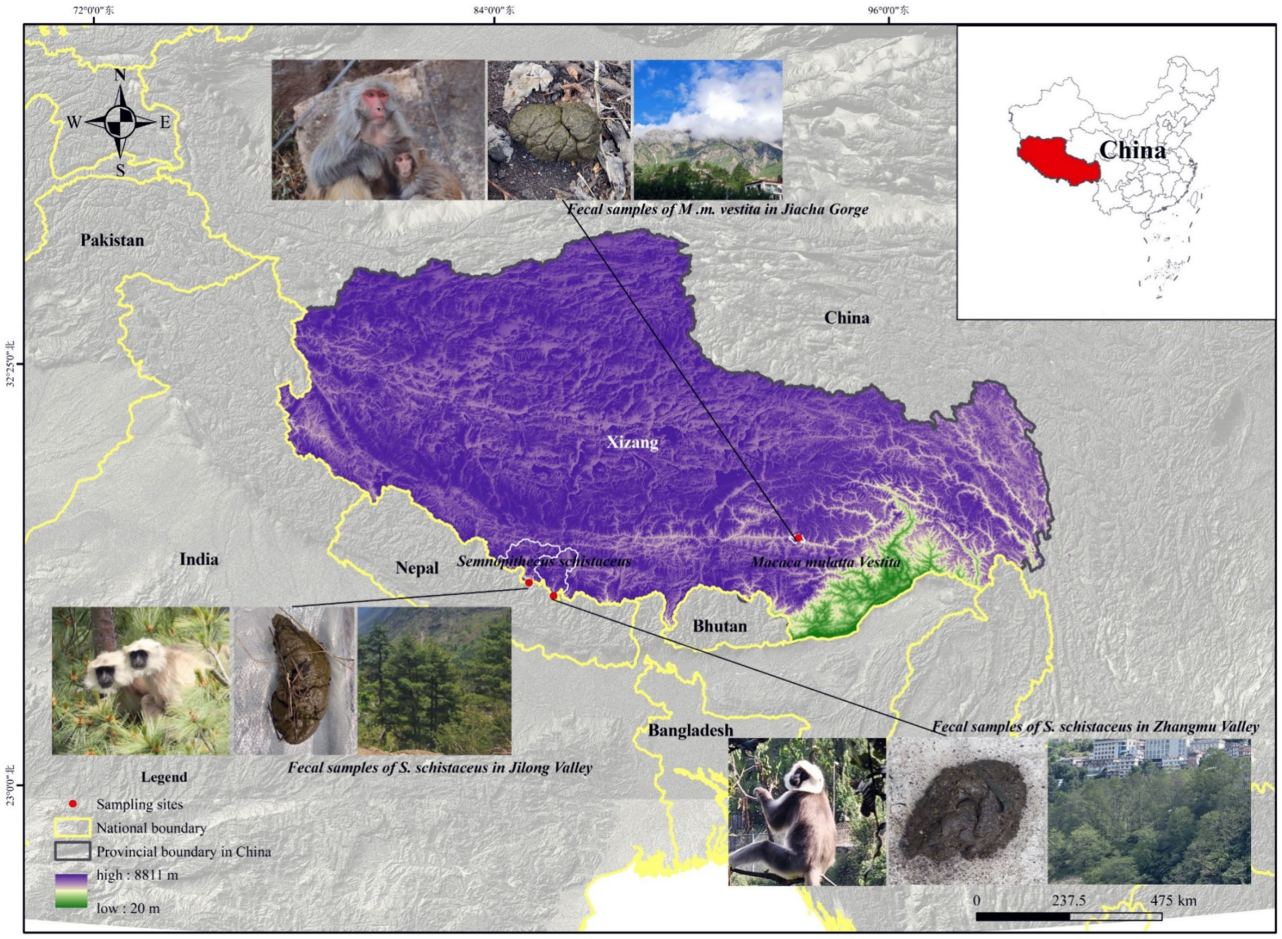
The study sites for the Himalayan langurs (*S. schistaceus*) and Xizang macaques (*M. m. vestita*) populations.

This study was conducted in Jilong Valley, Jilong County, Xizang, China (84°35′ −86°20′E, 28°3′ −29°3’N), Zhangmu Valley, Nielamu County, Xizang, China (85°27′ −86°37′E, 27°55′ −29°08’N), and Jiacha Gorge on the Yarlung Zangbo River, Xizang, China (92°11′ −92°36′E, 29°7′ −29°20’N) (Figure 1). The Himalayan langurs (*S. schistaceus*) and Xizang macaques (*M. m. vestita*) are both key protected wildlife species in China. This protection aims to safeguard their populations and habitats from threats such as habitat destruction and poaching. In May 2022, fecal samples from Himalayan langurs and Xizang macaques, as well as soil samples, were collected. Specifically, 18 fecal samples (LMJLG) were collected from Himalayan langurs in Jilong Valley at approximately 2,350 meters, 11 fecal samples (LMZMG) and 6 soil samples (Soil) were obtained from Himalayan langurs in Zhangmu Valley at approximately 2,000–2,500 meters, and 11 fecal samples (RM) from Xizang macaques and 2 soil samples (Soil) were obtained from Jiacha Gorge at approximately 3,500 meters. Fresh fecal samples were collected into sterile centrifuge tubes, sealed, labeled, and stored in portable dry ice containers until transported to the laboratory for final storage at −80°C. Detailed sample information used in this study is provided in Supplementary Tables S1, S2. It is noted that soil and fecal samples were collected from locations within the region that are representative of the typical ecological characteristics. While the samples were not taken from identical spots, this was a deliberate choice aimed at capturing the broader environmental context of the study area. The selected sites adequately represent the wide-ranging environments in which the primates reside, providing a more holistic view of the influence of environmental factors on gut microbiota. This design allows us to assess the impact of regional environmental heterogeneity, rather than limiting the study to the microenvironment of a single sampling point. In addition, we did not measure the physicochemical parameters of the habitat environment, as our primary focus was on the composition and functional adaptations of the microbiome. Despite this limitation, we believe that our study provides valuable insights into microbiome dynamics even without these environmental data. However, incorporating these measurements in future research could provide a more comprehensive understanding.

### Total DNA extraction

The total DNA was extracted from fresh fecal and soil samples using two different kits optimized for each sample type: the QIAamp DNA Stool Mini Kit (Qiagen) for fecal samples, and the DNeasy PowerSoil Pro Kit (Qiagen) for soil samples. These kits were selected for their effectiveness in extracting high-quality DNA from the complex matrices typical of fecal and soil samples, thereby ensuring sufficient yield and purity for downstream sequencing applications.

### The full-length 16S rRNA gene sequencing and analysis

The full-length 16S rRNA gene, covering the V1-V9 regions, was amplified using the universal primers 27F (5′-TACGGYTACCTTGTTACGACTT-3′) and 1492R (5′-AGAGTTTGATCMTGGCTCAG-3′). These primers are widely recognized for their broad specificity across bacterial taxa, enabling a comprehensive assessment of microbial diversity. PCR reactions were performed in a 25 μL volume. Thermal cycling conditions included an initial denaturation at 94°C for 5 min, followed by 35 cycles of denaturation at 94°C for 30 s, annealing at 55°C for 30 s, and extension at 72°C for 1 min, with a final extension at 72°C for 10 min. The amplified 16S rRNA gene products were then sequenced on the Illumina MiSeq platform for paired-end sequencing.

Paired-end reads were preprocessed using Trimmomatic software (Bolger et al., 2014). This step involved detecting and removing ambiguous bases (N) and low-quality sequences with an average quality score below 20 using a sliding window trimming approach. Parameters were set to trim bases once the average quality in a 4 bp window dropped below Q20. After trimming, the paired-end reads were assembled using FLASH software (Reyon et al., 2012) with the following parameters: a minimum overlap of 10 bp, a maximum overlap of 200 bp, and a maximum mismatch rate of 20%. This assembly step ensured that only high-confidence sequence pairs were retained for further analysis. Additional sequence denoising was performed by filtering out reads with ambiguous or homologous sequences, or those shorter than 200 bp. Reads with at least 75% of bases above Q20 were retained for further analysis using the QIIME software (version 1.8.0) (Caporaso et al., 2010). Potential chimeric sequences were detected and removed using VSEARCH (Rognes et al., 2016), which is known for its effective chimera detection capabilities. The clean reads were then subjected to primer sequence removal and clustered into operational taxonomic units (OTUs) with a 97% similarity cutoff using VSEARCH software. A representative read from each OTU was selected using the QIIME package and annotated against the Silva database (Version 123) using the RDP classifier with a confidence threshold of 70% (Wang et al., 2007). This taxonomic assignment process provided a comprehensive overview of the microbial composition in each sample.

For the visualization of microbial community structures in the four sample groups (LMJLG, LMZMG, RM, and Soil), several comprehensive analyses were performed. NMDS (Non-metric Multidimensional Scaling) and PCoA (Principal Coordinates Analysis) were used to visualize differences in microbial composition based on Unweighted UniFrac distances, highlighting distinct clustering patterns among the groups. Venn diagrams were generated to display the shared and unique operational taxonomic units (OTUs) across the groups. Relative abundances of dominant phyla and genera were visualized using heatmaps to illustrate differences in microbial communities at the taxonomic level. LEfSe (Linear Discriminant Analysis Effect Size) analysis was conducted to identify significantly different taxa among the groups, with results visualized using LDA (Linear Discriminant Analysis) bar plots for taxa with LDA values greater than 4.0 (*p* < 0.05). These visualizations provided a comprehensive understanding of the microbial diversity and composition influenced by geographic and environmental factors.

### Metagenomic sequencing and analysis

Metagenomic sequencing was performed using the Illumina HiSeq-PE150 platform (Sample details are provided in Supplementary Table S2). The raw data for each metagenome were approximately 50G, resulting in a cumulative total of approximately 1500G of raw reads for the 30 newly collected metagenomes in this study. Additionally, 12 metagenomic datasets included in this analysis were obtained from a previously published study (Li et al., 2023), and their data sizes are consistent with those reported in that publication. The raw data underwent processing using a methodology aligned with our previous research (Xia et al., 2022; Li et al., 2023). The procedure encompassed the following steps: Filtering Raw Reads: Cutadapt (Martin, 2011) was employed to filter the raw reads, utilizing the default parameters. Host Contamination Removal: BWA was used to eliminate host contamination. Assembly: MEGAHIT (Li et al., 2015) was applied to assemble the clean reads, following default parameters with a minimum contig length of 500 bp. Prediction of Coding Regions: MetaGeneMark (Zhu et al., 2010) was utilized to predict the coding regions within the contigs. Clustering and Unigene Formation: The predicted coding regions were clustered using CD-HIT (Fu et al., 2012) with specified parameters (identity ≥95% and overlap ≥90%), resulting in the formation of unigenes. Abundance Estimation: The abundance of unigenes was estimated using transcripts per million (TPM) values, based on the number of aligned reads, facilitated by bowtie2 (Langmead and Salzberg, 2012). Taxonomic and Functional Annotation: Diamond (Buchfink et al., 2015) was employed to align the unigenes against the NCBI micro-Non-Redundant (NR) database, which encompasses bacteria, fungi, archaea, and viruses. Only bacterial unigenes were retained for subsequent analyses.

For ARGs, identified genes were blasted against the Antibiotic Resistance Genes Database (CARD) using SARG2.0, and the putative ARG sequences were further blasted against the NCBI NR database to determine their microbiome sources (Yin et al., 2018). Customized Perl scripts were used to calculate the abundance (TPM) of ARG types and subtypes for each metagenome. For Carbohydrate-Active EnZymes (CAZymes), genes were annotated using the Carbohydrate-Active EnZymes database (CAZy) (Cantarel et al., 2009). Identified CAZyme genes were blasted against the CAZy database to classify them into respective enzyme families. KEGG pathway analysis involved annotating unigenes against the KEGG database and mapping them to KEGG Orthology terms (Buchfink et al., 2015; Kanehisa et al., 2023).

Principal Component Analysis (PCA) and distance analysis were employed to visualize clustering patterns for bacterial taxonomy, ARGs, CAZymes, and KEGG pathways among the groups. Additionally, ANOVA tests were conducted to analyze the data. Before performing ANOVA, we verified the assumptions of normality and homogeneity of variances using the Shapiro–Wilk test and Levene’s test, respectively. The results of these tests confirmed that the data met the necessary assumptions for ANOVA, thereby ensuring the validity of the test outcomes. LEfSe analysis identified significant differences in bacterial abundance, ARG abundance, CAZyme profiles, and KEGG pathway enrichment among the groups, providing insights into the functional adaptations of the gut microbiota to different environmental conditions. The relative abundance of ARGs was visualized using Circos diagrams to illustrate connections between the top 15 bacterial genera and the top 15 ARGs among the groups. Additionally, Circos diagrams were also utilized to show the connections between the top 15 bacterial genera and the top 15 CAZymes, providing a detailed view of the carbohydrate metabolism capabilities within the microbiomes.

## Results and discussion

### Impact of geographic location and soil environment on gut bacterial communities of Himalayan langurs and Xizang macaques

**Figure 2:**
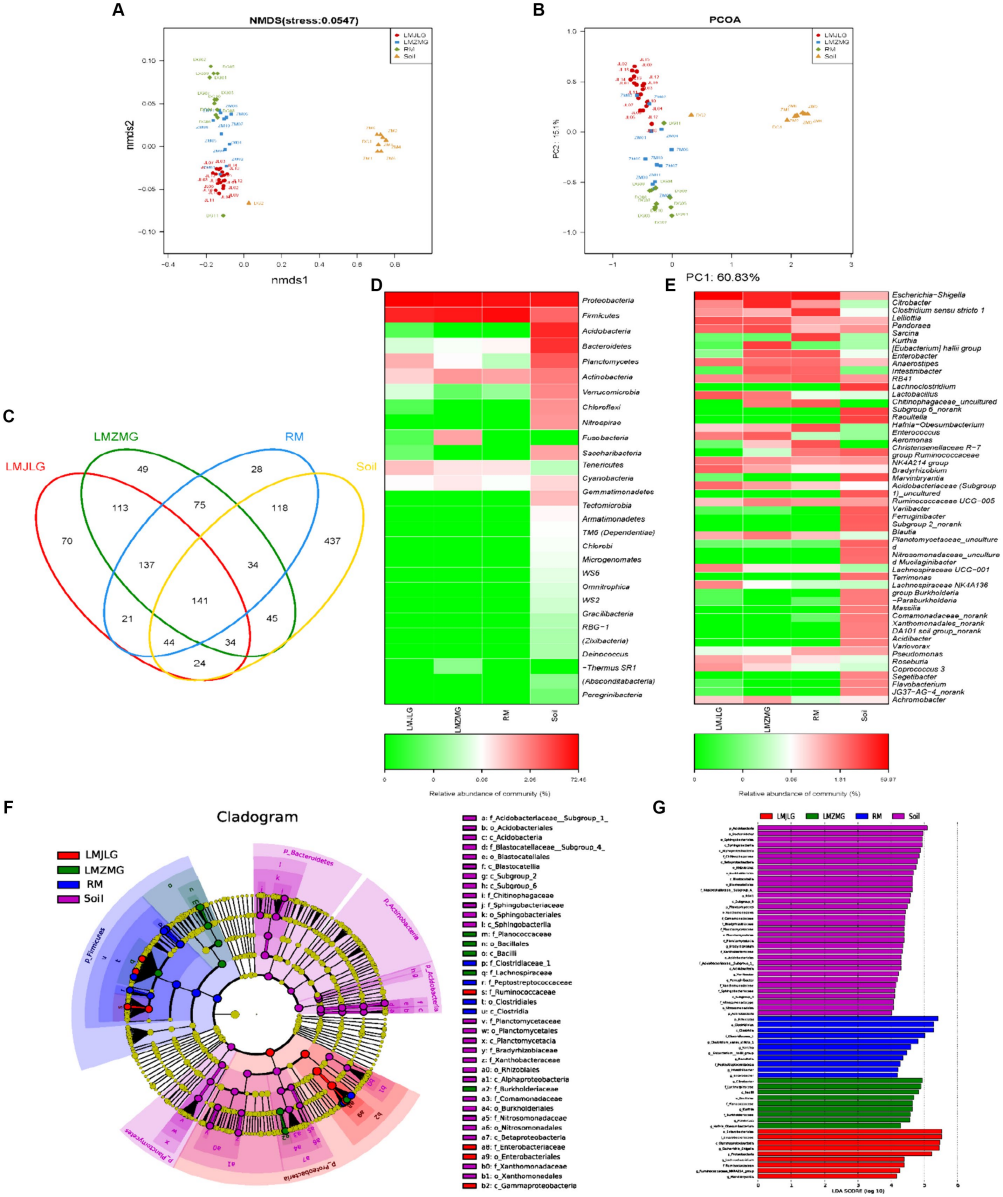
The composition of the gut microbiome among four groups (LMJLG, LMZMG, RM, and Soil). **(A)** NMDS analysis based on unweighted UniFrac distance. **(B)** PCoA analysis based on unweighted UniFrac distance. **(C)** Composition of OTUs in LMJLG, LMZMG, RM, and Soil. **(D)** Relative abundance of the dominant phyla in LMJLG, LMZMG, RM, and Soil. **(E)** Relative abundance of the dominant genera in LMJLG, LMZMG, RM, and Soil. **(F)** LEfSe analysis showing significant differences in the abundance of gut microbiomes among LMJLG, LMZMG, RM, and Soil. **(G)** Histogram of LDA values of gut differential species in LMJLG, LMZMG, RM, and Soil (LDA value >4.0, *p* < 0.05).

Our study aimed to understand how different geographic locations within the Himalayas influenced the gut microbial communities of Himalayan langurs and Xizang Macaques. We conducted comprehensive 16S rRNA sequencing on fecal samples collected from Himalayan langurs in Jilong Valley (LMJLG) and Zhangmu Valley (LMZMG), and from Xizang Macaques in Jiacha Gorge (RM), with Soil samples from Zhangmu Valley and Jiacha Gorge served as environmental controls (Soil). NMDS analysis (Figure 2A) and PCoA analysis (Figure 2B) revealed distinct clustering of microbial communities based on different geographic and altitudinal factors. Both analyses demonstrated clear separation among the four microbial groups (LMJLG, LMZMG, RM, and Soil), with a particularly notable distance between the gut microbiota groups and the soil microbiota group. Soil-derived microorganisms played a significant role in shaping the gut microbial community of hosts, and the ingestion of soil by soil-dwelling animals facilitated the colonization of these microorganisms in the gut (Tyrrell et al., 2019; Li et al., 2021; Fu et al., 2023). Some animals, including reptiles, birds, and non-human primates, exhibited geophagy (soil-eating behavior), which allowed them to supplement mineral nutrients or detoxify ingested food in their digestive tracts (Young et al., 2011; Lee et al., 2014; Pebsworth et al., 2018; Monaco et al., 2019). Additionally, it was reported that Nepalese gray langurs (*S. schistaceus*) might obtain sodium through geophagy (Monaco et al., 2019). Another evidence also showed that the gut microbiome of Xizang Macaques was more similar to that of their plant diet than to the soil microbiome (Sun et al., 2021). Similarly, our study results indicated a great dissimilarity between the gut microbiota of langurs and macaques and the soil microbiota, suggesting that the impact of the soil environment on their gut microbiota was not as pronounced. Additionally, the distance between LMJLG and LMZMG samples was smaller compared to the distance between LMJLG and RM, and LMJLG shared more OTUs with LMZMG (113 OTUs) (Figure 2C), indicating a greater similarity in the gut microbiota between the langurs from Jilong Valley and Zhangmu Valley.

Heatmap analyses at the phylum level (Figure 2D) and genus level (Figure 2E) revealed differences in microbial abundance among the four groups. The gut microbiota of both Himalayan langurs (LMJLG and LMZMG) and Xizang Macaques (RM) were dominated by Proteobacteria and Firmicutes. Proteobacteria were most abundant in RM (52.34%), whereas Firmicutes were more prevalent in LMJLG (72.45%) and LMZMG (59.11%). At the genus level, *Escherichia-Shigella* predominated in LMJLG, LMZMG, and RM, with a particularly high relative abundance in LMJLG (56.18%). Additionally, LMZMG samples showed a significant presence of *Citrobacter* (18.77%), *Kurthia* (9.86%), and *Pandoraea* (6.73%), while RM samples were enriched with *Clostridium sensu stricto 1* (13.05%) and *Sarcina* (10.20%). Notably, *Lelliottia* was more abundant in the langur groups (LMJLG: 5.90% and LMZMG: 4.61%) compared to the macaque group RM (0.52%). The Soil samples displayed a more diverse microbial community, with Proteobacteria (39.20%), Acidobacteria (23.73%), and Bacteroidetes (18.82%) being the most abundant phyla. At the genus level, soil samples contained higher levels of *RB41* (7.51%), *Chitinophagaceae_uncultured* (6.57%), *Subgroup 6_norank* (6.37%), and *Bradyrhizobium* (4.37%). LEfSe analysis (Figures 2F,G) was conducted to identify the taxa most likely to explain the differences between the microbial communities in the four groups. At the genus level, *Escherichia-Shigella*, *Lachnoclostridium*, *Ruminococcaceae_NK4A214_group*, and *Marvinbryantia* were more abundant in LMJLG. *Citrobacter*, *Kurthia*, *Pandoraea*, and *Hafnia_Obesumbacterium* were more enriched in LMZMG. *Clostridium_sensu_stricto_1*, *Sarcina*, *Eubacterium_hallii_group*, *Raoultella*, *Intestinibacter*, and *Enterobacter* were more prevalent in RM. Lastly, *Bradyrhizobium* was more prominent in Soil. These findings suggest that geographic location and environmental factors might influence the gut microbiota of Himalayan langurs and Xizang Macaques, which we speculate could be due to specific environmental and dietary conditions (Hu et al., 2021; Li et al., 2023). This quantification provided significant microbial biomarkers that differentiated each group and offered insights into the ecological and functional roles of these taxa.

### Geographic variation in metagenomic profiles of langurs and macaques

**Figure 3:**
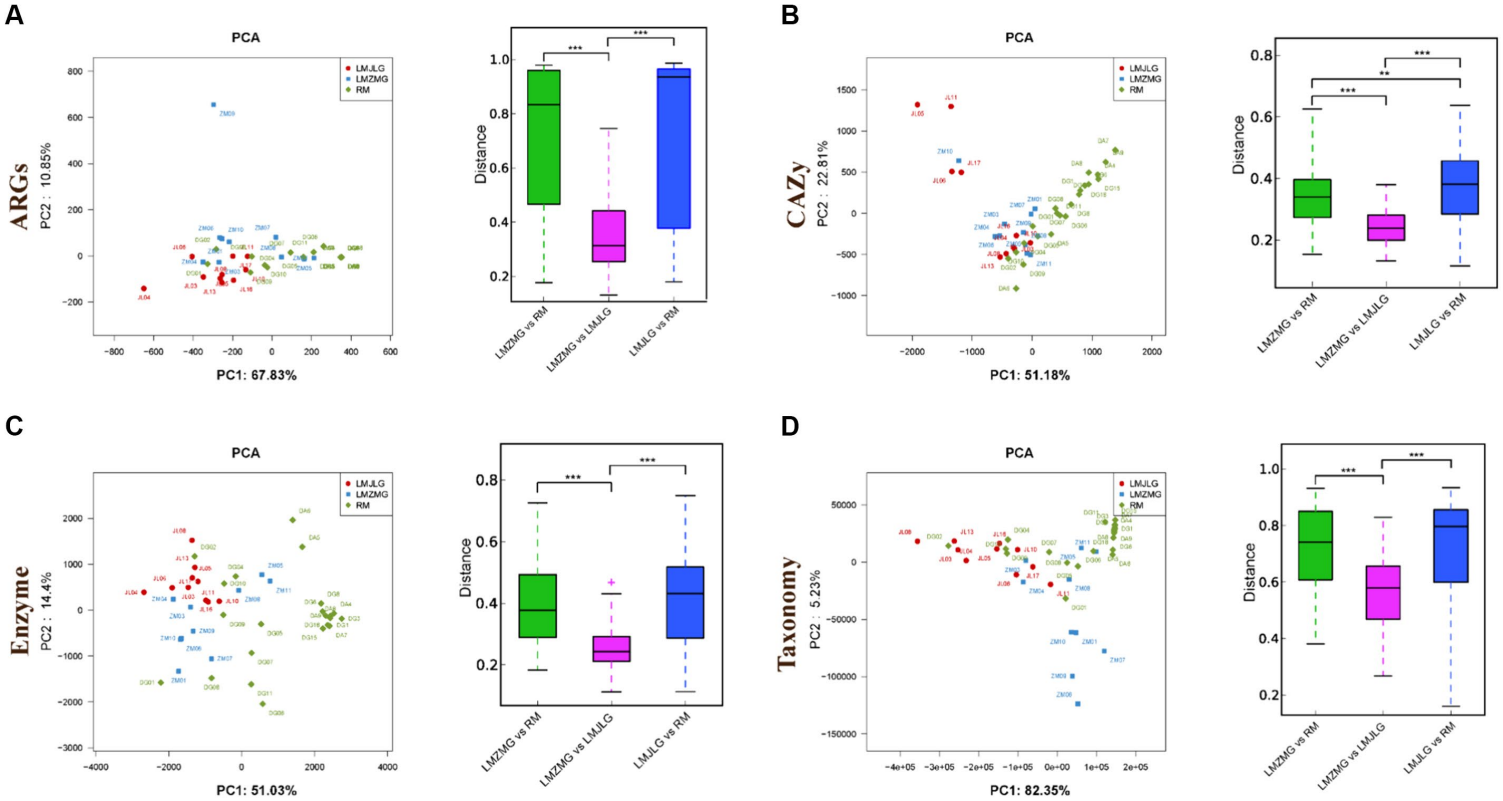
The beta diversity of gut microbiome in three groups (LMJDL, LMZMG, and RM). The PCA and distance analysis of LMJDL, LMZMG, and RM in **(A)** ARGs, **(B)** CAZymes, **(C)** Kegg enzymes, and **(D)** phylogenetic taxonomy. Significant correlations are indicated by black stars: **p < 0.01, ***p < 0.001.

We analyzed 42 metagenomes from langurs and macaques collected from three distinct geographic locations. The PCA plots (Figure 3) demonstrated distinct clustering patterns for antibiotic resistance genes (ARGs), carbohydrate-active enzymes (CAZy), KEGG enzyme functions, and taxonomic profiles among the three groups (LMJLG, LMZMG, and RM). Distance analysis (Figure 3) further revealed that the pairwise distances for ARGs, CAZy, KEGG enzyme functions, and taxonomic profiles between LMZMG-RM and LMJLG-RM were significantly greater compared to the distances between LMZMG-LMJLG. This indicated a closer similarity in these profiles between the two langur groups (LMJLG and LMZMG) than between the langurs and the macaques (RM).

### Comparative analysis of ARGs profiles in gut microbiota of Himalayan langurs and Xizang macaques

**Figure 4:**
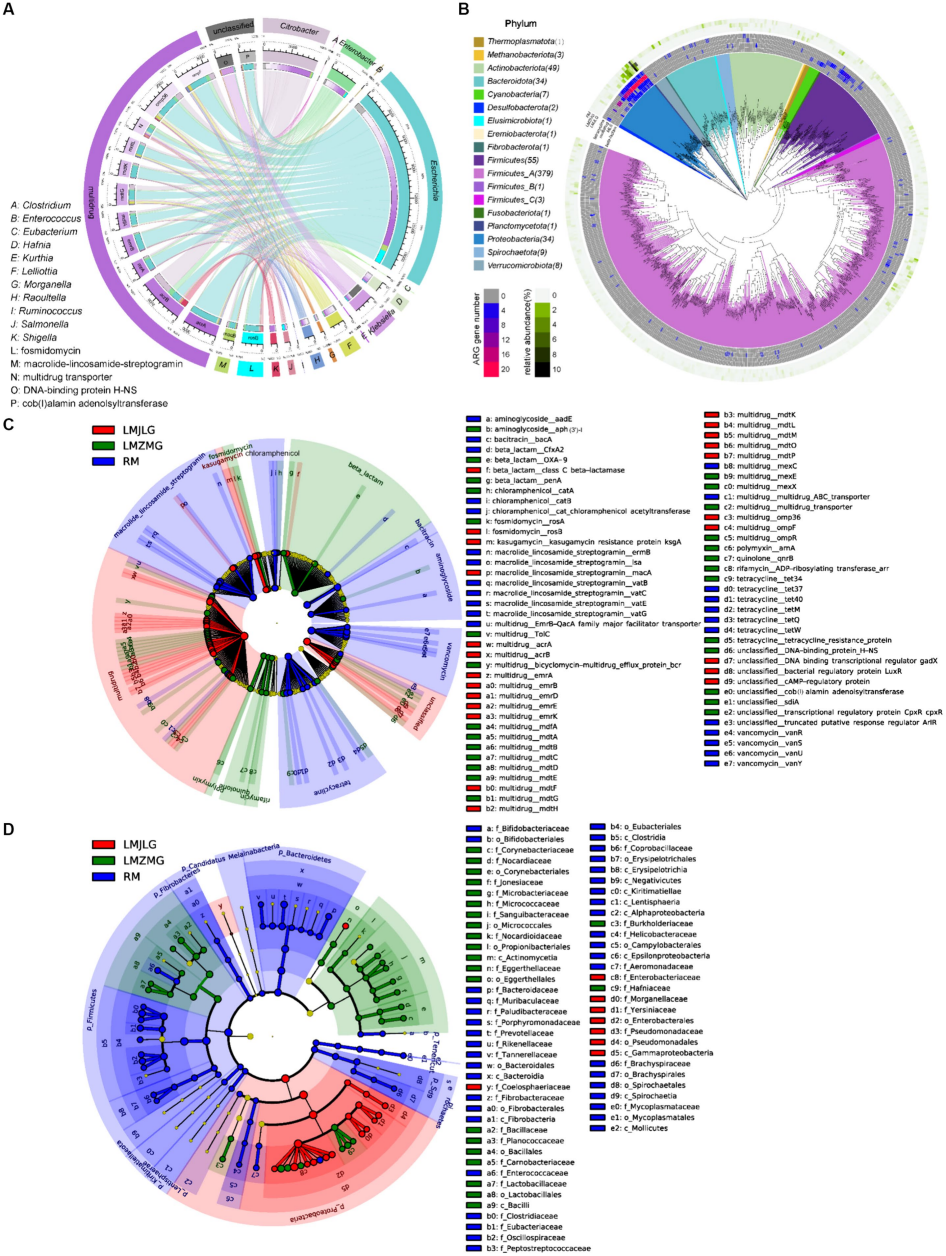
Comprehensive analysis of ARGs in the metagenomes of the three groups. **(A)** Circos diagram showing connections between the top 15 bacterial genera and the top 15 ARGs among the three groups. The length of the bars on the outer and inner rings represents the percentage and relative abundance of each gut microbiome group (genus level), as well as ARG type and subtype in their respective sections. Each genus is represented by a specific ribbon color, and the width of each ribbon indicates the proportion of each genus in each ARG type and subtype. **(B)** Distribution of the 590 non-redundant metagenome-assembled genomes (MAGs) from 42 metagenomes. Phylogenetic analysis of these 590 MAGs (coverage >80%, contamination rate < 10%) is displayed in the central circle as maximum-likelihood trees created using the MAGs. The outer circle heatmap shows the relative abundance of each bin (MAG) in each group or the gene numbers in each specific ARG category (tetracycline, multidrug, MLS, and beta-lactam). **(C)** LEfSe analysis identifying significant differences in the abundance of ARGs among LMJLG, LMZMG, and RM. **(D)** LEfSe analysis identifying significant differences in the abundance of gut microbiomes among LMJLG, LMZMG, and RM.

Through the association of the top 15 bacterial genera and ARG subtypes (Figure 4A), we found that multidrug resistance genes (acrA, acrB, emrA, emrB, mdfA, mdtG, mdtK, mdtL, transporter, omp36, ompF), fosmidomycin resistance gene (rosB), macrolide-lincosamide-streptogramin (MLS_macB) resistance gene, and unclassified_cob_I_alamin_adenolsyltransferase were primarily associated with *Escherichia*, *Citrobacter*, and *Enterobacter*, with *Escherichia* being particularly prominent. Additionally, the unclassified DNA-binding protein H-NS was related to *Klebsiella*. Previous studies have reported that Histone-like nucleoid-structuring protein (H-NS) inhibited the expression of virulence factors such as type-3 pili and capsule in *K. pneumoniae* (Ares et al., 2016; Ares et al., 2017). Furthermore, H-NS could influence *K. pneumoniae*’s ability to acquire resistance genes to imipenem, a beta-lactam antibiotic, by inhibiting the activity of the CRISPR-Cas system (Lin et al., 2016).

We obtained 590 high-quality metagenome-assembled genomes (MAGs) from 42 metagenomes of langurs and macaques collected from three different geographic locations. Among these, approximately 15 MAGs (*Lelliottia*, *Citrobacter europaeus*, *Escherichia coli*, *Lelliottia amnigena*, *Serratia liquefaciens_A*, *Citrobacter gillenii*, *Klebsiella_A michiganensis*, *Raoultella ornithinolytica*, *Enterobacter_D kobei_A*, *Atlantibacter*, *Morganella morganii_A*, *Morganella morganii_B*, *Morganella psychrotolerans_B*, *Morganella morganii*, *Morganella*) were found to commonly contain genes encoding four typical ARGs: multidrug, tetracycline, beta-lactam, and MLS, especially multidrug resistance genes (Figure 4B). Specifically, the LMJLG samples contained the highest number of *E. coli* and its associated multidrug resistance genes (Figure 3B, Supplementary Tables S3, S4). Previous studies have reported that *E. coli* isolates from captive non-human primates in zoos exhibit a high diversity of antibiotic resistance phenotypes, carrying multiple antibiotic resistance genes and integrons, indicating that NHPs have substantial potential for the spread of multidrug resistance (Zhu et al., 2021). Additionally, the LEfSe analysis (Figure 4C and Supplementary Figure S1) identified specific ARGs that were significantly enriched in each group. LMJLG samples showed higher levels of ARGs associated with multidrug (65.34%) resistance, while LMZMG samples were enriched in ARGs related to beta-lactam (5.00%), polymyxin (1.19%), quinolone (0.98%), and rifamycin (0.58%). RM samples exhibited a broader range of ARGs, including those for MLS (5.94%), tetracycline (6.89%), vancomycin (3.82%), bacitracin (4.88%), aminoglycoside (1.88%), and chloramphenicol (1.52%) resistance.

The global rise in antibiotic use has created a major public health challenge: the widespread presence of ARGs in microbial communities. These ARGs are found in diverse environments, including soil, aquatic systems (oceans, lakes, groundwater), and the gastrointestinal tracts of humans and animals (Torres et al., 2020a; Zheng et al., 2021; Wu et al., 2023). The persistence and spread of antimicrobial resistance are further complicated by the often diverse or unknown origins of these resistance genes. Therefore, antimicrobial resistance is common in wildlife species even without direct antibiotic exposure (Torres et al., 2020a; Torres et al., 2020b). Additionaly, environmental and ecological conditions in different geographical areas also play a crucial role in the development of bacterial communities and drug resistance profiles. Previous evidence found in giant pandas suggests that different habitat ecology (captive or wild) and wild geographic distribution patterns promote the presence and composition of ARGs in giant panda gut of different geographic populations (Hu et al., 2021; Deng et al., 2024). Our study identified a significant prevalence of *Escherichia*, particularly *E. coli*, and associated multidrug resistance (MDR) genes in the LMJLG samples (Figure 4D and Supplementary Figure S2). This finding suggests that these langurs in Jilong valley may serve as a potential reservoir of MDR genes. *E. coli* is a versatile pathogen commonly found in the gut microbiota of humans and animals and plays a crucial role in MDR (Poirel et al., 2018; Foster-Nyarko and Pallen, 2022). It can rapidly acquire and disseminate resistance genes through horizontal gene transfer mechanisms facilitated by mobile genetic elements (MGEs) such as plasmids, transposons, and integrons (Rozwadowski and Gawel, 2022). This capability is essential for its adaptability and survival under selective pressures like antibiotic treatments. Moreover, MDR *E. coli* often coexists with various virulence factors, including adhesins, toxins, and iron acquisition systems, which enhance its pathogenicity and ability to cause infections (Kaper et al., 2004; Sarowska et al., 2019). Studies have shown that certain *E. coli* strains, particularly uropathogenic *E. coli* (UPEC), are particularly adept at acquiring resistance while maintaining or enhancing their virulence, complicating treatment efforts (Rozwadowski and Gawel, 2022). The high levels of ARGs associated with *E. coli* in the LMJLG samples from in Jilong valley may indicate active gene exchange and a robust potential for resistance gene dissemination in this region. The high prevalence of MDR genes in the LMJLG langur samples may arise from unique ecological factors and selective pressures, such as contaminated water sources, proximity to human activities, and interactions with other wildlife (Kang et al., 2021; Catalano et al., 2022). These conditions facilitate the survival and spread of resistant *E. coli* strains. Therefore, monitoring wildlife, such as langurs, is crucial for understanding and managing environmental ARGs contamination. Effective conservation strategies must incorporate wildlife’s role in transmitting resistant pathogens to mitigate resistance sources. This approach not only protects wildlife health but also contributes to global public health efforts against MDR pathogens. Understanding the ecological and environmental drivers behind the prevalence of ARGs can inform targeted interventions to control their dissemination, ultimately safeguarding both human and animal health (Redpath et al., 2013; Laborda et al., 2022).

### Comparative analysis of CAZyme profiles in gut microbiota of Himalayan langurs and Xizang macaques

**Figure 5:**
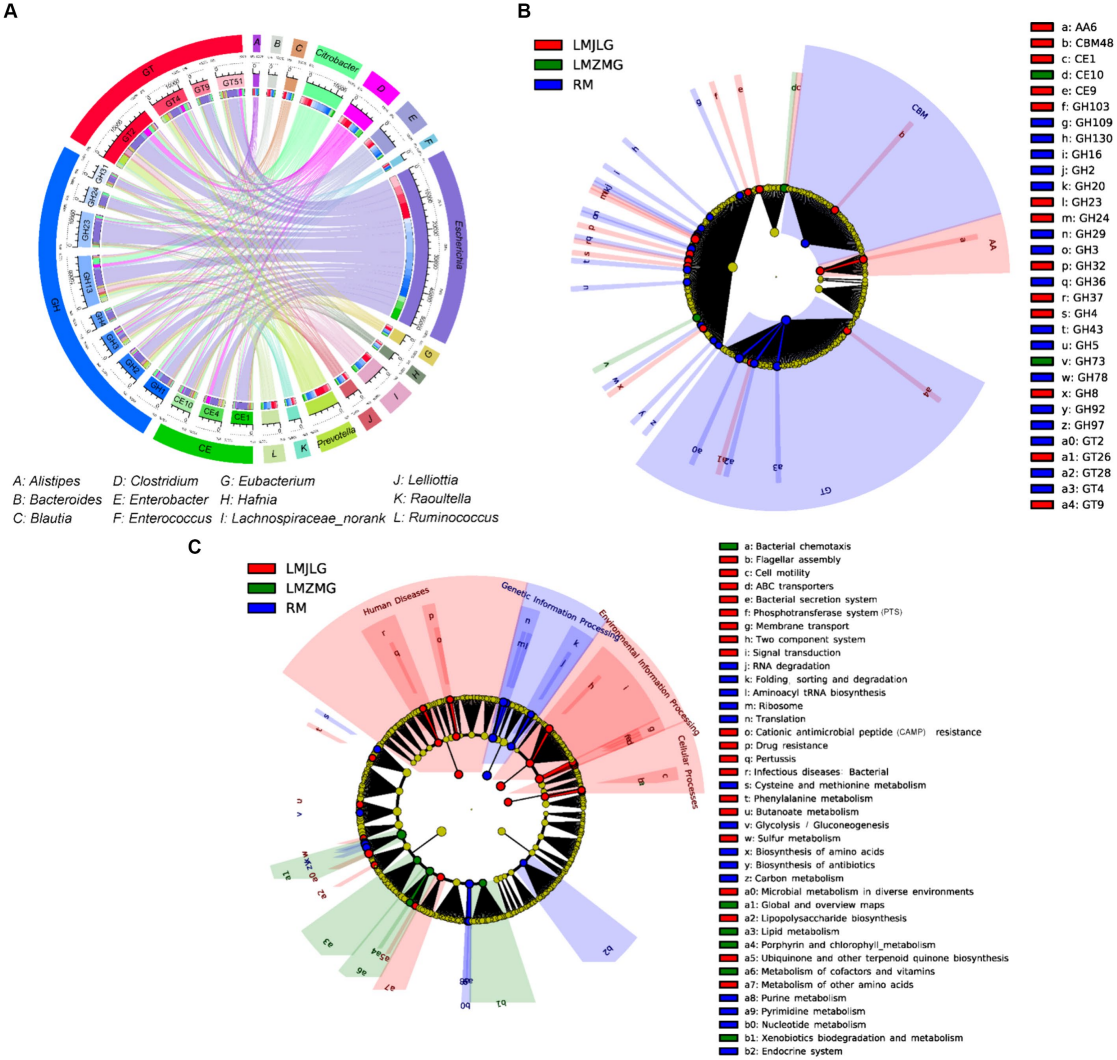
Comprehensive analysis of CAZyme genes in the metagenomes of three groups. **(A)** Circos Diagram of Connections of Top15 Bacteria and Top15 CAZyme among three groups. The length of the bars on the outer rings and inner rings represented the percentage and relative abundance of each gut microbiome group (genus level), and CAZyme in their respective sections, respectively. Each genus was represented by a specific ribbon color, and the width of each ribbon showed the proportion of each genus in each CAZyme. **(B)** LEfSe analysis was used to determine the significant difference in the abundance of CAZyme among LMJDL, LMZMG, and RM. **(C)** LEfSe analysis was used to determine the significant difference in the abundance of Kegg pathway among LMJDL, LMZMG, and RM.

Figure 5A illustrated the association between the top 15 bacterial genera and the top 15 CAZyme subtypes, revealing that specific CAZyme genes were predominantly linked to certain bacterial genera. For instance, glycoside hydrolases (GH), glycosyl transferases (GT), and carbohydrate esterases (CE) genes were mainly associated with *Escherichia*, *Citrobacter*, *Prevotella*, *Clostridium*, and *Enterobacter*. Among these, *Escherichia* demonstrated the strongest association with all top 15 CAZy genes (GH1, GH2, GH3, GH4, GH13, GH23, GH24, GH31, GT2, GT4, GT9, GT51, CE1, CE4, and CE10), indicating its significant role in carbohydrate metabolism within these microbiomes in LMHLG, LMZMG and RM. Notably, the LEfSe analysis (Figure 5B) identified that GH103, GT26, CE1, CBM48, GH4, GH8, CE9, GT9, GH24, GH23, AA6, GH32, and GH37 were significantly abundant in LMJLG, while GH73 and CE10 were particularly abundant in LMZMG. In contrast, glycosyl transferases (GT), carbohydrate-binding modules (CBM), GH109, GT28, GH2, GH3, GH5, GH16, GH92, GH97, GH130, GH20, GH29, GH43, GT4, GH78, GH36, and GT2 were enriched in RM. This distribution underscored the unique metabolic capabilities of the microbial communities in these groups.

The KEGG pathway (Figure 5C and Supplementary Figure S3) analysis further revealed that carbohydrate metabolism pathways were differentially enriched among the groups. Specifically, the Glycolysis/Gluconeogenesis pathway was abundant in RM, indicating a reliance on fundamental energy production and glucose metabolism processes (Rigoulet et al., 2020). Glycolysis/fermentation and oxidative phosphorylation are the two most major pathways of host cell energy metabolism (Rigoulet et al., 2020). The significant enrichment of glycoside hydrolases (GHs), glycosyl transferases (GTs), and carbohydrate-binding modules (CBMs) in the RM group suggests that these enzymes may play a crucial role in the glycolysis and fermentation processes of this group. Whereas, the Butanoate metabolism pathway was significantly enriched in LMJLG, suggesting active short-chain fatty acid metabolism, which is often linked to gut health and microbial fermentation processes (Singh et al., 2023). Some gut microbes contribute to the degradation of dietary fibers into oligosaccharides and monosaccharides, which are then fermented to produce short-chain fatty acids (SCFAs) such as acetate, propionate, and butyrate (Blaak et al., 2020; Fu et al., 2022; Portincasa et al., 2022). *Escherichia* and *Pseudomonas*, which were enriched in the LMJLG group (Figure 4D), demonstrated the ability to degrade cellulose and other polysaccharides (Huang et al., 2012; Baltazar-Díaz et al., 2022; Su et al., 2024), potentially facilitating the synthesis of butyric acid and other SCFAs. Furthermore, studies have shown that CAZymes, including the GH3, GH5, GH39, GH43, GH48, GH53, and AA4 families, may be involved in butyrate metabolism (Wang et al., 2013; Liu et al., 2021). This finding is consistent with our study, which identified an enrichment of GH (GH103, GH4, GH8, GH24, GH23, GH32, GH37) and AA family enzymes in the LMJLG group.

The primary diet of Himalayan langurs consists of fiber-rich leaves, which may lead to the enrichment of carbohydrate-active enzymes and the butanoate metabolism pathway in the LMJLG population (Mo et al., 2023). Notably, despite both LMJLG and LMZMG being langur populations, significant differences in their carbohydrate metabolism pathways were observed. Specifically, the butanoate metabolism pathway was significantly enriched only in LMJLG, while LMZMG showed no significant enrichment of carbohydrate enzyme-related metabolic pathways. This disparity may be due to geographical heterogeneity. Although current research cannot precisely explain these heterogeneities, it is hypothesized that these differences are closely related to varying geographic locations and their corresponding environmental conditions. Additionally, the Himalayan langurs of LMJLG and LMZMG inhabit mid-altitude regions, whereas the Xizang macaques (RM) reside at high altitudes. The significant enrichment of the glycolysis/gluconeogenesis pathway in Xizang macaques indicates a higher demand for glucose metabolism and basic energy production, likely due to the harsh conditions at high altitudes that limit food availability and utilization efficiency (Fan et al., 2020; Zhou et al., 2022). In such environments, rapid and efficient energy production mechanisms are crucial for survival (Fan et al., 2020; Feijó et al., 2020). We hypothesize that the high-fiber diet of langurs in Jilong Valley may promote the proliferation of butyrate-producing microbes and associated GHs. Different dietary patterns could be a key factor driving the observed differences in microbial communities and enzyme enrichments between LMJLG and RM. This highlights the complex interplay between diet, microbiome composition, and metabolic output.

To further validate these hypotheses, future research should focus on the following areas: first, detailed analyses of how different geographic locations and environmental conditions affect the microbiome and metabolic pathways of langurs and Xizang macaques. Second, comparative studies of the dietary compositions of different populations, particularly fiber content and types, to understand their specific impacts on the microbiome and metabolic products (Hamaker and Tuncil, 2014; Cronin et al., 2021). Additionally, employing genomics and metabolomics approaches to identify and verify the functions and mechanisms of key carbohydrate-active enzymes and metabolic pathways in different populations (Jendoubi, 2021; Wörheide et al., 2021). Finally, controlled experiments simulating various dietary and environmental pressures should be conducted to observe their effects on microbiome composition and metabolic function (Allaband et al., 2019; Ecklu-Mensah et al., 2022). Through these studies, a more comprehensive understanding of the intricate relationships between environment, diet, and microbiome can be achieved, thereby providing scientific evidence for the conservation and management of these primate populations.

## Conclusion

This study used 16S rRNA and metagenomic sequencing to reveal unique clustering patterns in the gut microbiota of Himalayan langurs (*S. schistaceus*) and Xizang macaques (*M. m. vestita*) from different geographic locations. The soil environment had little influence on their gut microbiota. The gut microbiomes of langurs from Jilong Valley (LMJLG) and Zhangmu Valley (LMZMG) were more similar to each other than to those of macaques from Jiacha Gorge (RM). A high prevalence of *E. coli* and multidrug resistance genes in LMJLG langurs suggests they could be reservoirs for these bacteria, likely due to unique ecological factors and selective pressures. Significant differences in carbohydrate-active enzymes and KEGG pathway functions were found between groups. Langurs from LMJLG showed enrichment in cellulolytic enzymes and butyrate metabolism, likely due to their high-fiber diet, while macaques from higher altitudes in Jiacha Gorge showed enrichment in glycolysis/gluconeogenesis pathways, reflecting their need for fundamental energy production. These findings highlight the critical role of geographic location and environmental factors in shaping the gut microbiota of Himalayan langurs and Xizang macaques. These insights contribute to our understanding of the ecological and functional roles of gut microbiota in these primates and emphasize the importance of considering geographic and environmental contexts in microbial ecology studies. Meanwhile, effective protection and management strategies can be formulated based on this study results.

## Data Availability

The original contributions presented in the study are publicly available. This data can be found here: https://www.ncbi.nlm.nih.gov/, accession numbers PRJNA1150390 and PRJNA1151203.
