# Supplementary material for: Geographic and environmental impacts on gut microbiome in Himalayan langurs (Semnopithecus schistaceus) and Xizang macaques (Macaca mulatta vestita)
Source: Front Microbiol. 2024 Sep 4;15:1452101. doi: 10.3389/fmicb.2024.1452101 (PMC11408304; doi:10.3389/fmicb.2024.1452101)
Supplement: Supplementary file 1 [file Table_1.DOCX]

# Supplementary information

## **Table S1** 48 datasets of 16S rRNA sequencing in this study

| **Group** | **16S ID** | **Species** | **Sampling time** | **Sampling site** | **Longitude** | **Latitude** | **Altitude (m)** |
| --- | --- | --- | --- | --- | --- | --- | --- |
| LMJLG | JL01 | *S. schistaceus* | May 2022 | Jilong County | 28.31417 | 85.3388 | 2352 |
| LMJLG | JL02 | *S. schistaceus* | May 2022 | Jilong County | 28.31417 | 85.3388 | 2352 |
| LMJLG | JL03 | *S. schistaceus* | May 2022 | Jilong County | 28.31417 | 85.3388 | 2352 |
| LMJLG | JL04 | *S. schistaceus* | May 2022 | Jilong County | 28.31417 | 85.3388 | 2352 |
| LMJLG | JL05 | *S. schistaceus* | May 2022 | Jilong County | 28.31417 | 85.3388 | 2352 |
| LMJLG | JL06 | *S. schistaceus* | May 2022 | Jilong County | 28.31417 | 85.3388 | 2352 |
| LMJLG | JL07 | *S. schistaceus* | May 2022 | Jilong County | 28.31417 | 85.3388 | 2352 |
| LMJLG | JL08 | *S. schistaceus* | May 2022 | Jilong County | 28.31417 | 85.3388 | 2352 |
| LMJLG | JL09 | *S. schistaceus* | May 2022 | Jilong County | 28.31417 | 85.3388 | 2352 |
| LMJLG | JL10 | *S. schistaceus* | May 2022 | Jilong County | 28.31417 | 85.3388 | 2352 |
| LMJLG | JL11 | *S. schistaceus* | May 2022 | Jilong County | 28.31417 | 85.3388 | 2352 |
| LMJLG | JL12 | *S. schistaceus* | May 2022 | Jilong County | 28.31417 | 85.3388 | 2352 |
| LMJLG | JL13 | *S. schistaceus* | May 2022 | Jilong County | 28.31417 | 85.3388 | 2352 |
| LMJLG | JL14 | *S. schistaceus* | May 2022 | Jilong County | 28.31417 | 85.3388 | 2352 |
| LMJLG | JL15 | *S. schistaceus* | May 2022 | Jilong County | 28.31417 | 85.3388 | 2352 |
| LMJLG | JL16 | *S. schistaceus* | May 2022 | Jilong County | 28.31417 | 85.3388 | 2352 |
| LMJLG | JL17 | *S. schistaceus* | May 2022 | Jilong County | 28.31417 | 85.3388 | 2352 |
| LMJLG | JL18 | *S. schistaceus* | May 2022 | Jilong County | 28.31417 | 85.3388 | 2352 |
| LMZMG | ZM01 | *S. schistaceus* | May 2022 | Zhangmu County | 27.96821 | 85.96927 | 1986 |
| LMZMG | ZM02 | *S. schistaceus* | May 2022 | Zhangmu County | 27.96821 | 85.96927 | 1986 |
| LMZMG | ZM03 | *S. schistaceus* | May 2022 | Zhangmu County | 27.96821 | 85.96927 | 1986 |
| LMZMG | ZM04 | *S. schistaceus* | May 2022 | Zhangmu County | 27.96821 | 85.96927 | 1986 |
| LMZMG | ZM05 | *S. schistaceus* | May 2022 | Zhangmu County | 27.96821 | 85.96927 | 1986 |
| LMZMG | ZM06 | *S. schistaceus* | May 2022 | Zhangmu County | 27.96821 | 85.96927 | 1986 |
| LMZMG | ZM07 | *S. schistaceus* | May 2022 | Zhangmu County | 27.98863 | 85.98865 | 2450 |
| LMZMG | ZM08 | *S. schistaceus* | May 2022 | Zhangmu County | 27.98863 | 85.98865 | 2450 |
| LMZMG | ZM09 | *S. schistaceus* | May 2022 | Zhangmu County | 27.98863 | 85.98865 | 2450 |
| LMZMG | ZM10 | *S. schistaceus* | May 2022 | Zhangmu County | 28.01984 | 85.98539 | 2563 |
| LMZMG | ZM11 | *S. schistaceus* | May 2022 | Zhangmu County | 28.01984 | 85.98539 | 2563 |
| RM | DG01 | *M. m. vestita* | May 2022 | Jiacha Gorge | 29.22368 | 92.34563 | 3579 |
| RM | DG02 | *M. m. vestita* | May 2022 | Jiacha Gorge | 29.22368 | 92.34563 | 3579 |
| RM | DG03 | *M. m. vestita* | May 2022 | Jiacha Gorge | 29.22368 | 92.34563 | 3579 |
| RM | DG04 | *M. m. vestita* | May 2022 | Jiacha Gorge | 29.22368 | 92.34563 | 3579 |
| RM | DG05 | *M. m. vestita* | May 2022 | Jiacha Gorge | 29.22368 | 92.34563 | 3579 |
| RM | DG06 | *M. m. vestita* | May 2022 | Jiacha Gorge | 29.22368 | 92.34563 | 3579 |
| RM | DG07 | *M. m. vestita* | May 2022 | Jiacha Gorge | 29.25963 | 92.40114 | 3540 |
| RM | DG08 | *M. m. vestita* | May 2022 | Jiacha Gorge | 29.25963 | 92.40114 | 3540 |
| RM | DG09 | *M. m. vestita* | May 2022 | Jiacha Gorge | 29.25963 | 92.40114 | 3540 |
| RM | DG10 | *M. m. vestita* | May 2022 | Jiacha Gorge | 29.25963 | 92.40114 | 3540 |
| RM | DG11 | *M. m. vestita* | May 2022 | Jiacha Gorge | 29.25963 | 92.40114 | 3540 |
| Soil | ZM1 | Soil | May 2022 | Zhangmu County | 27.96821 | 85.96927 | 1986 |
| Soil | ZM2 | Soil | May 2022 | Zhangmu County | 27.96821 | 85.96927 | 1986 |
| Soil | ZM3 | Soil | May 2022 | Zhangmu County | 27.96821 | 85.96927 | 1986 |
| Soil | ZM4 | Soil | May 2022 | Zhangmu County | 27.97477 | 85.97416 | 2014 |
| Soil | ZM5 | Soil | May 2022 | Zhangmu County | 27.99388 | 85.97656 | 2926 |
| Soil | ZM6 | Soil | May 2022 | Zhangmu County | 28.01984 | 85.98539 | 2563 |
| Soil | DG1 | Soil | May 2022 | Jiacha Gorge | 29.22368 | 92.34563 | 3579 |
| Soil | DG2 | Soil | May 2022 | Jiacha Gorge | 29.25963 | 92.40114 | 3540 |

## **Table S2** 42 of datasets metagenomic in this study

| **Group** | **Meta ID** | **Species** | **Sampling time** | **Sampling site** | **Longitude** | **Latitude** | **Altitude (m)** | **References** |
| --- | --- | --- | --- | --- | --- | --- | --- | --- |
| LMJLG | JL03 | *S. schistaceus* | May 2022 | Jilong County | 85.33879867 | 28.31416571 | 2352 | \ |
| LMJLG | JL04 | *S. schistaceus* | May 2022 | Jilong County | 85.33879867 | 28.31416571 | 2352 | \ |
| LMJLG | JL05 | *S. schistaceus* | May 2022 | Jilong County | 85.33879867 | 28.31416571 | 2352 | \ |
| LMJLG | JL06 | *S. schistaceus* | May 2022 | Jilong County | 85.33879867 | 28.31416571 | 2352 | \ |
| LMJLG | JL08 | *S. schistaceus* | May 2022 | Jilong County | 85.33879867 | 28.31416571 | 2352 | \ |
| LMJLG | JL10 | *S. schistaceus* | May 2022 | Jilong County | 85.33879867 | 28.31416571 | 2352 | \ |
| LMJLG | JL11 | *S. schistaceus* | May 2022 | Jilong County | 85.33879867 | 28.31416571 | 2352 | \ |
| LMJLG | JL13 | *S. schistaceus* | May 2022 | Jilong County | 85.33879867 | 28.31416571 | 2352 | \ |
| LMJLG | JL16 | *S. schistaceus* | May 2022 | Jilong County | 85.33879867 | 28.31416571 | 2352 | \ |
| LMJLG | JL17 | *S. schistaceus* | May 2022 | Jilong County | 85.33879867 | 28.31416571 | 2352 | \ |
| LMZMG | ZM01 | *S. schistaceus* | May 2022 | Zhangmu County | 85.96927241 | 27.96821451 | 1986 | \ |
| LMZMG | ZM03 | *S. schistaceus* | May 2022 | Zhangmu County | 85.96927241 | 27.96821451 | 1986 | \ |
| LMZMG | ZM04 | *S. schistaceus* | May 2022 | Zhangmu County | 85.96927241 | 27.96821451 | 1986 | \ |
| LMZMG | ZM05 | *S. schistaceus* | May 2022 | Zhangmu County | 85.96927241 | 27.96821451 | 1986 | \ |
| LMZMG | ZM06 | *S. schistaceus* | May 2022 | Zhangmu County | 85.96927241 | 27.96821451 | 1986 | \ |
| LMZMG | ZM07 | *S. schistaceus* | May 2022 | Zhangmu County | 85.98865309 | 27.98863043 | 2450 | \ |
| LMZMG | ZM08 | *S. schistaceus* | May 2022 | Zhangmu County | 85.98865309 | 27.98863043 | 2450 | \ |
| LMZMG | ZM09 | *S. schistaceus* | May 2022 | Zhangmu County | 85.98865309 | 27.98863043 | 2450 | \ |
| LMZMG | ZM10 | *S. schistaceus* | May 2022 | Zhangmu County | 85.98538931 | 28.01984081 | 2563 | \ |
| LMZMG | ZM11 | *S. schistaceus* | May 2022 | Zhangmu County | 85.98538931 | 28.01984081 | 2563 | \ |
| RM | DG01 | *M. m. vestita* | May 2022 | Jiacha Gorge | 92.34562885 | 29.2236751 | 3579 | \ |
| RM | DG02 | *M. m. vestita* | May 2022 | Jiacha Gorge | 92.34562885 | 29.2236751 | 3579 | \ |
| RM | DG04 | *M. m. vestita* | May 2022 | Jiacha Gorge | 92.34562885 | 29.2236751 | 3579 | \ |
| RM | DG05 | *M. m. vestita* | May 2022 | Jiacha Gorge | 92.34562885 | 29.2236751 | 3579 | \ |
| RM | DG06 | *M. m. vestita* | May 2022 | Jiacha Gorge | 92.34562885 | 29.2236751 | 3579 | \ |
| RM | DG07 | *M. m. vestita* | May 2022 | Jiacha Gorge | 92.40113743 | 29.2596266 | 3540 | \ |
| RM | DG08 | *M. m. vestita* | May 2022 | Jiacha Gorge | 92.40113743 | 29.2596266 | 3540 | \ |
| RM | DG09 | *M. m. vestita* | May 2022 | Jiacha Gorge | 92.40113743 | 29.2596266 | 3540 | \ |
| RM | DG10 | *M. m. vestita* | May 2022 | Jiacha Gorge | 92.40113743 | 29.2596266 | 3540 | \ |
| RM | DG11 | *M. m. vestita* | May 2022 | Jiacha Gorge | 92.40113743 | 29.2596266 | 3540 | \ |
| RM | DG1 | *M. m. vestita* | May 2020 | Jiacha Gorge | 92°11′–92°36′ E | 29°7′–29°20′ N | 3200~4752 | (Li et al., 2023) |
| RM | DG3 | *M. m. vestita* | May 2020 | Jiacha Gorge | 92°11′–92°37′ E | 29°7′–29°21′ N | 3200~4753 | (Li et al., 2023) |
| RM | DG6 | *M. m. vestita* | May 2020 | Jiacha Gorge | 92°11′–92°38′ E | 29°7′–29°22′ N | 3200~4754 | (Li et al., 2023) |
| RM | DG8 | *M. m. vestita* | May 2020 | Jiacha Gorge | 92°11′–92°39′ E | 29°7′–29°23′ N | 3200~4755 | (Li et al., 2023) |
| RM | DG15 | *M. m. vestita* | May 2020 | Jiacha Gorge | 92°11′–92°40′ E | 29°7′–29°24′ N | 3200~4756 | (Li et al., 2023) |
| RM | DG18 | *M. m. vestita* | May 2020 | Jiacha Gorge | 92°11′–92°41′ E | 29°7′–29°25′ N | 3200~4757 | (Li et al., 2023) |
| RM | DA4 | *M. m. vestita* | May 2020 | Jiacha Gorge | 92°11′–92°42′ E | 29°7′–29°26′ N | 3200~4758 | (Li et al., 2023) |
| RM | DA5 | *M. m. vestita* | Aug 2020 | Jiacha Gorge | 92°11′–92°43′ E | 29°7′–29°27′ N | 3200~4759 | (Li et al., 2023) |
| RM | DA6 | *M. m. vestita* | Aug 2020 | Jiacha Gorge | 92°11′–92°44′ E | 29°7′–29°28′ N | 3200~4760 | (Li et al., 2023) |
| RM | DA7 | *M. m. vestita* | Aug 2020 | Jiacha Gorge | 92°11′–92°45′ E | 29°7′–29°29′ N | 3200~4761 | (Li et al., 2023) |
| RM | DA8 | *M. m. vestita* | Aug 2020 | Jiacha Gorge | 92°11′–92°46′ E | 29°7′–29°30′ N | 3200~4762 | (Li et al., 2023) |
| RM | DA9 | *M. m. vestita* | Aug 2020 | Jiacha Gorge | 92°11′–92°47′ E | 29°7′–29°31′ N | 3200~4763 | (Li et al., 2023) |

## **Table S3** Relative abundance of Top 20 MAGs in three groups

| Gene_ID | Taxology | LMJLG | LMZMG | RM |
| --- | --- | --- | --- | --- |
| DG09.Bin13 | *s__Escherichia coli* | 25.77124 | 9.051027 | 8.333684 |
| ZM09.Bin72 | *s__Citrobacter gillenii* | 0.172339 | 6.638691 | 0.148163 |
| JL11.Bin39 | *s__Citrobacter europaeus* | 2.209356 | 4.029354 | 0.498867 |
| JL04.Bin28 | *g__Lelliottia* | 3.968686 | 2.263707 | 0.127787 |
| ZM03.Bin44 | *s__Lelliottia amnigena* | 2.755545 | 2.873419 | 0.563288 |
| JL17.Bin1 | *s__Hafnia alvei* | 1.008882 | 1.989793 | 0.034096 |
| DG01.Bin32 | *s__Raoultella ornithinolytica* | 0.48382 | 0.338321 | 1.607145 |
| DG02.Bin12 | *s__Enterobacter_D kobei_A* | 0.674676 | 0.630747 | 1.107047 |
| DG05.Bin29 | *g__Anaerobutyricum* | 0.065308 | 0.115412 | 1.137712 |
| DA7.Bin2 | *g__RC9* | 7.90E-05 | 9.92E-04 | 1.25E+00 |
| ZM01.Bin47 | *s__Hafnia proteus* | 0.133706 | 0.997129 | 0.01433 |
| DG3.Bin23 | *g__UBA1232* | 3.70E-05 | 3.17E-03 | 1.14E+00 |
| JL17.Bin53 | *s__Serratia liquefaciens_A* | 0.932556 | 0.122344 | 0.078578 |
| JL03.Bin60 | *g__Atlantibacter* | 0.908877 | 0.088938 | 0.036815 |
| JL03.Bin33 | *g__UBA7160* | 0.764269 | 0.17567 | 0.005268 |
| DG05.Bin25 | *s__Klebsiella_A michiganensis* | 0.190536 | 0.557743 | 0.182427 |
| JL17.Bin45 | *s__Rahnella variigena* | 0.621372 | 0.190258 | 0.076133 |
| ZM07.Bin13 | *g__Kurthia* | 0.001231 | 0.850243 | 0.001471 |
| DA4.Bin19 | *g__Prevotella* | 9.67E-05 | 4.99E-02 | 7.43E-01 |
| DA5.Bin44 | *g__Anaerostipes* | 0.001746 | 0.0647 | 0.698643 |

## **Table S4** Gene Numbers for Beta-lactam, Macrolide-Lincosamide-Streptogramin (MLS), Multidrug, and Tetracycline Resistance in the Top 30 MAGs Across Three Groups

| **Gene_ID** | **Taxonomy** | **Beta-lactam** | **MLS** | **Multidrug** | **Tetracycline** |
| --- | --- | --- | --- | --- | --- |
| JL04.Bin28 | *g__Lelliottia* | 3 | 2 | 35 | 2 |
| JL11.Bin39 | *s__Citrobacter europaeus* | 4 | 3 | 33 | 2 |
| DG09.Bin13 | *s__Escherichia coli* | 1 | 2 | 37 | 1 |
| ZM03.Bin44 | *s__Lelliottia amnigena* | 2 | 2 | 32 | 3 |
| JL17.Bin53 | *Js__Serratia liquefaciens_A* | 3 | 2 | 28 | 4 |
| ZM09.Bin72 | *s__Citrobacter gillenii* | 3 | 2 | 30 | 1 |
| DG05.Bin25 | *s__Klebsiella_A michiganensis* | 3 | 2 | 29 | 2 |
| JL17.Bin45 | *s__Rahnella variigena* | 2 | 0 | 28 | 2 |
| DG01.Bin32 | *s__Raoultella ornithinolytica* | 1 | 2 | 26 | 2 |
| DG02.Bin12 | *s__Enterobacter_D kobei_A* | 2 | 2 | 26 | 1 |
| JL03.Bin60 | *g__Atlantibacter* | 1 | 2 | 24 | 2 |
| JL17.Bin1 | *s__Hafnia alvei* | 2 | 0 | 25 | 2 |
| ZM01.Bin47 | *s__Hafnia proteus* | 2 | 0 | 23 | 2 |
| JL16.Bin70 | *s__Morganella morganii_A* | 2 | 2 | 17 | 3 |
| ZM01.Bin40 | *s__Morganella morganii_B* | 2 | 2 | 16 | 3 |
| ZM11.Bin26 | *s__Morganella psychrotolerans_B* | 1 | 3 | 15 | 4 |
| ZM08.Bin66 | *s__Morganella morganii* | 2 | 2 | 16 | 2 |
| JL05.Bin9 | *g__Morganella* | 1 | 2 | 16 | 2 |
| JL16.Bin3 | *s__Proteus hauseri* | 0 | 2 | 13 | 1 |
| JL11.Bin26 | *s__Pseudomonas_E sp000282515* | 1 | 0 | 14 | 0 |
| JL06.Bin15 | *g__Pseudomonas_E* | 1 | 0 | 13 | 0 |
| JL17.Bin48 | *s__Pseudomonas_E sp001269815* | 1 | 0 | 13 | 0 |
| ZM03.Bin60 | *g__Ralstonia* | 2 | 0 | 7 | 0 |
| DG06.Bin4 | *s__Enterococcus_B faecium* | 0 | 3 | 5 | 0 |
| ZM07.Bin83 | *s__Aeromonas bestiarum* | 3 | 0 | 3 | 1 |
| ZM06.Bin52 | *s__Enterococcus_D sp002850555* | 0 | 1 | 5 | 0 |
| ZM08.Bin51 | *s__Enterococcus_B hirae* | 0 | 1 | 5 | 0 |
| ZM11.Bin33 | *s__Enterococcus_B pernyi* | 0 | 1 | 5 | 0 |
| DG06.Bin56 | *s__Enterococcus_B durans* | 0 | 1 | 5 | 0 |
| ZM06.Bin49 | *s__Lactococcus lactis* | 0 | 1 | 2 | 1 |

##
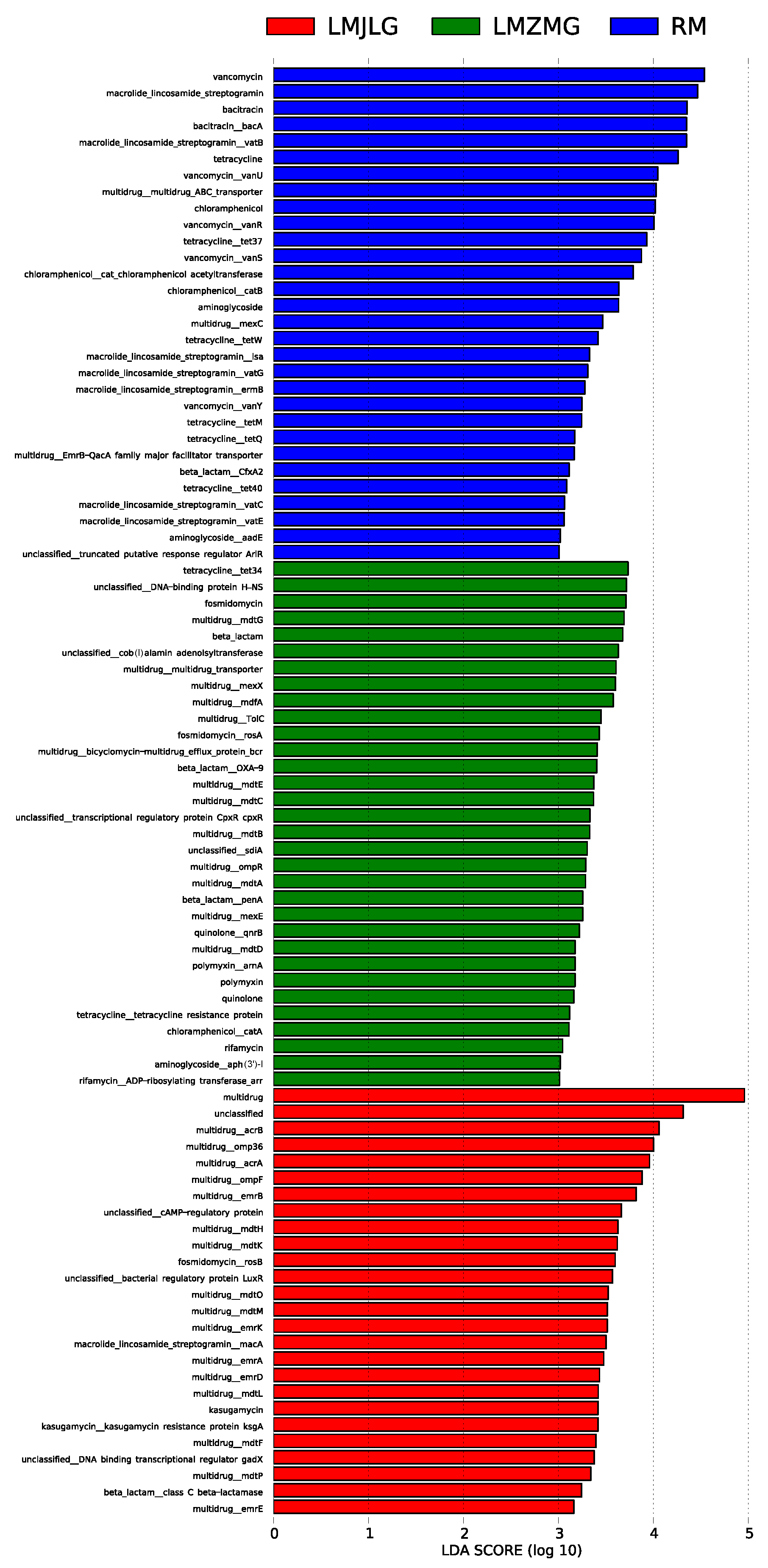
**Figure S1** Histogram of LDA values of gut differential ARGs in LMJLG, LMZMG, and RM (LDA value > 3.0, p < 0.05).

## **Figure S2** Histogram of LDA values of gut differential gut bacteria in LMJLG, LMZMG, and RM (LDA value > 3.0, p < 0.05).


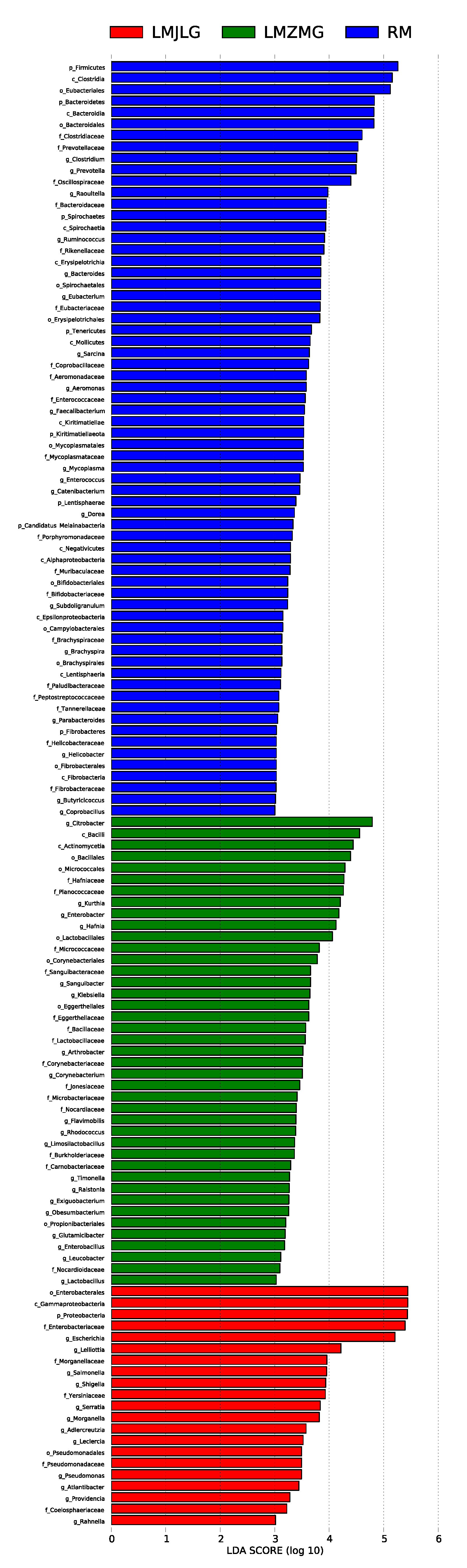


## **
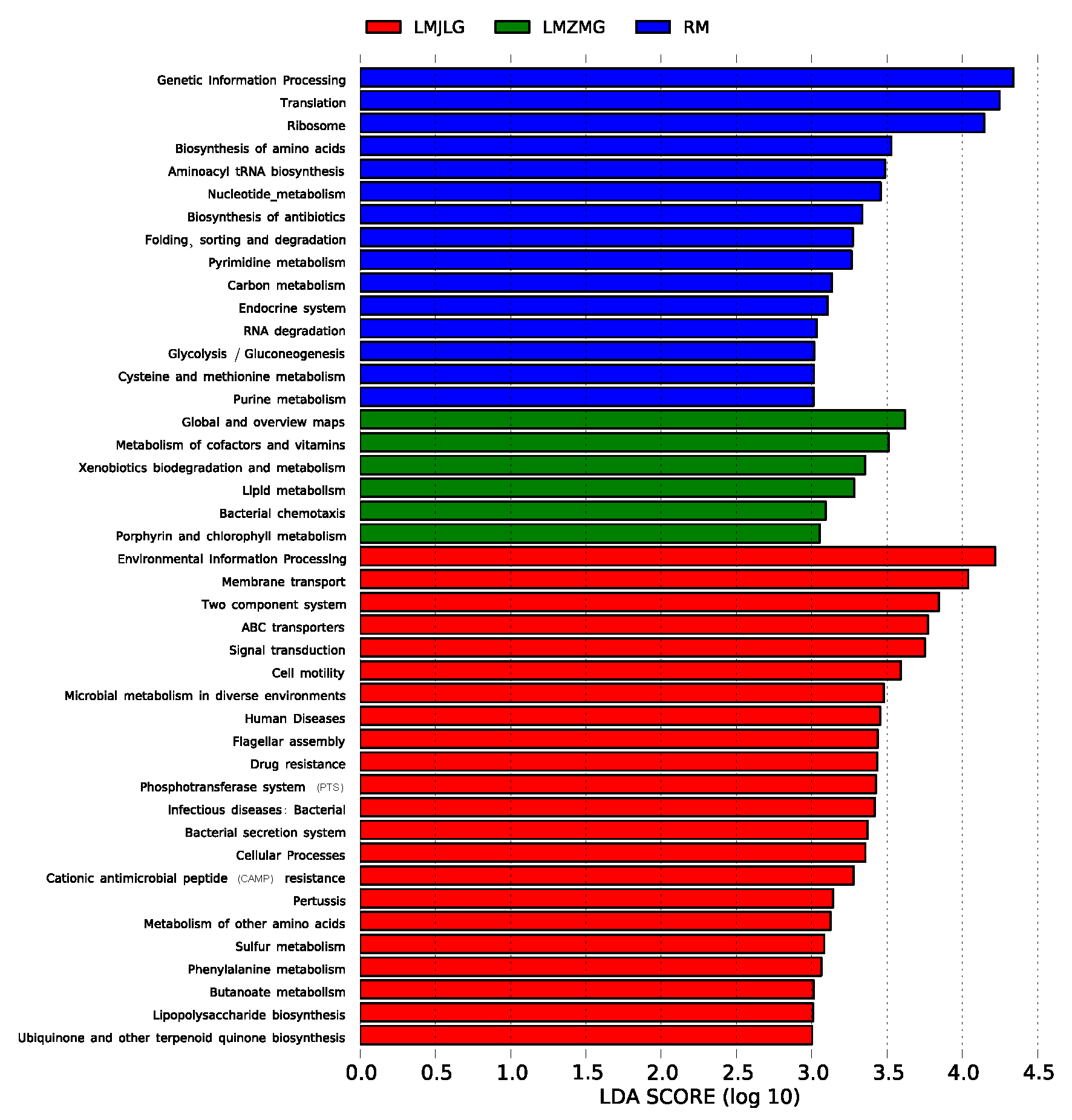
Figure S3** Histogram of LDA values of gut differential Kegg pathway in LMJLG, LMZMG, and RM (LDA value > 3.0, p < 0.05).

## **Perl scripts** for calculating the abundance (TPM) of ARG types and subtypes for each metagenome:

#!/usr/bin/perl -w

use strict;

use warnings;

my ($argAnno,$samOrder,$type,$geneProfile,$prefix);

my $opt;

while($opt = shift){

if($opt eq "-i"){

$argAnno = shift;

}elsif($opt eq "-r"){

$samOrder = shift;

}elsif($opt eq "-t"){

$type = shift;

}elsif($opt eq "-p"){

$geneProfile = shift;

}elsif($opt eq "-o"){

$prefix = shift;

}elsif($opt eq "-h"){

&usage;exit;

}else{

print STDERR "unknown parameter: $opt\n";

exit;

}

}

unless($argAnno and $samOrder and $geneProfile and $prefix){

&usage;

exit;

}

$type = "p" unless($type);

my (@order,%geneAbun,%type2gene,%subType2gene);

my %temp_used;

open INS,$samOrder or die "$!\n";

while(<INS>){

chomp;

next if(/^#/);

my @temp = split;

push @order,$temp[0] unless(exists $temp_used{$temp[0]});

$temp_used{$temp[0]} = 1;

}

close INS;

open ING,$geneProfile or die "$!\n";

my %temp_order;

while(<ING>){

chomp;

my @temp = split /\t/;

for(my $i = 1;$i < @temp;$i++){

if($. == 1){

$temp_order{$i} = $temp[$i];

}else{

$geneAbun{$temp_order{$i}}{$temp[0]} = $temp[$i];

}

}

}

close ING;

open INT,$argAnno or die "$!\n";

while(<INT>){

chomp;

my @temp = split /\t/;

next if(/^#/);

if($type eq "p" or $type eq "P"){

$temp[0] = $1 if($temp[0] =~ /(\S+)\_/);

}

$type2gene{$temp[1]} .= ",$temp[0]";

$subType2gene{$temp[2]} .= ",$temp[0]";

# $tax2gene{$temp[1]} .= ",$temp[0]" unless($temp[1] eq "NO-TaxID" or $temp[1] eq "NO-Tax");

}

close INT;

open OUT,"> $prefix.subType.txt" or die "$!\n";

print OUT "#SubType\t";

print OUT join "\t",@order;

print OUT "\n";

foreach my $subType(sort keys %subType2gene){

print OUT $subType;

$subType2gene{$subType} =~ s/\,//;

my @gene = split /\,/,$subType2gene{$subType};

my %tempAbun;

foreach my $sam(@order){

foreach my $g(@gene){

die "error: $g has no abundance in $sam!!!\n" unless(exists $geneAbun{$sam}{$g});

$tempAbun{$sam} += $geneAbun{$sam}{$g};

}

printf OUT "\t%.3f",$tempAbun{$sam};

}

print OUT "\n";

}

close OUT;

open OUB,"> $prefix.type.txt" or die "$!\n";

print OUB "#Type\t";

print OUB join "\t",@order;

print OUB "\n";

foreach my $type(sort keys %type2gene){

print OUB $type;

$type2gene{$type} =~ s/\,//;

my @gene = split /\,/,$type2gene{$type};

my %tempAbun;

foreach my $sam(@order){

foreach my $g(@gene){

die "error: $g has no abundance in $sam!!!\n" unless(exists $geneAbun{$sam}{$g});

$tempAbun{$sam} += $geneAbun{$sam}{$g};

}

printf OUB "\t%.3f",$tempAbun{$sam};

}

print OUB "\n";

}

close OUB;

sub usage{

print <<EOD

usage: perl $0 -i arg.anno.txt -r sam.order -t type[n|p] -p gene.profile -o prefix

-i arg annotation from 4-20.arg.anno.pl, required

-r sample order file, required

-t blast type[n|p],n:nucleotide p:protein,default p

-p gene profile, required

-o out prefix,required

EOD

}

# Reference

Li, D., Xia, W., Cui, X., Zhao, M., Huang, K., Wang, X., Shen, J., Chen, H., and Zhu, L. (2023). The putatively high-altitude adaptation of macaque monkeys: Evidence from the fecal metabolome and gut microbiome. Evolutionary Applications *16*, 1708-1720.
